# DNA Methylation Markers and Prediction Model for Depression and Their Contribution for Breast Cancer Risk

**DOI:** 10.3389/fnmol.2022.845212

**Published:** 2022-02-23

**Authors:** Ning Wang, Jing Sun, Tao Pang, Haohao Zheng, Fengji Liang, Xiayue He, Danian Tang, Tao Yu, Jianghui Xiong, Suhua Chang

**Affiliations:** ^1^Affective Disorder Department, Beijing Huilongguan Hospital, Beijing, China; ^2^Department of Biobank, Liaoning Cancer Hospital and Institute, Cancer Hospital of China Medical University, Shenyang, China; ^3^NHC Key Laboratory of Mental Health, National Clinical Research Center for Mental Disorders, Peking University Sixth Hospital, Peking University Institute of Mental Health, Chinese Academy of Medical Sciences Research Unit, Peking University, Beijing, China; ^4^State Key Laboratory of Space Medicine Fundamentals and Application, China Astronaut Research and Training Center, Beijing, China; ^5^Gastrointestinal Surgery Department, Beijing Hospital, Beijing, China; ^6^Department of Medical Imaging, Liaoning Cancer Hospital and Institute, Cancer Hospital of China Medical University, Shenyang, China; ^7^Deepome. Inc., Beijing, China; ^8^Lab of Epigenetics and Advanced Health Technology, Space Science and Technology Institute, Shenzhen, China

**Keywords:** major depressive disorder, DNA methylation, prediction model, breast cancer, mDI

## Abstract

**Background:**

Major depressive disorder (MDD) has become a leading cause of disability worldwide. However, the diagnosis of the disorder is dependent on clinical experience and inventory. At present, there are no reliable biomarkers to help with diagnosis and treatment. DNA methylation patterns may be a promising approach for elucidating the etiology of MDD and predicting patient susceptibility. Our overarching aim was to identify biomarkers based on DNA methylation, and then use it to propose a methylation prediction score for MDD, which we hope will help us evaluate the risk of breast cancer.

**Methods:**

Methylation data from 533 samples were extracted from the Gene Expression Omnibus (GEO) database, of which, 324 individuals were diagnosed with MDD. Statistical difference of DNA Methylation between Promoter and Other body region (SIMPO) score for each gene was calculated based on the DNA methylation data. Based on SIMPO scores, we selected the top genes that showed a correlation with MDD in random resampling, then proposed a methylation-derived Depression Index (mDI) by combining the SIMPO of the selected genes to predict MDD. A validation analysis was then performed using additional DNA methylation data from 194 samples extracted from the GEO database. Furthermore, we applied the mDI to construct a prediction model for the risk of breast cancer using stepwise regression and random forest methods.

**Results:**

The optimal mDI was derived from 426 genes, which included 245 positive and 181 negative correlations. It was constructed to predict MDD with high predictive power (AUC of 0.88) in the discovery dataset. In addition, we observed moderate power for mDI in the validation dataset with an OR of 1.79. Biological function assessment of the 426 genes showed that they were functionally enriched in Eph Ephrin signaling and beta-catenin Wnt signaling pathways. The mDI was then used to construct a predictive model for breast cancer that had an AUC ranging from 0.70 to 0.67.

**Conclusion:**

Our results indicated that DNA methylation could help to explain the pathogenesis of MDD and assist with its diagnosis.

## Introduction

Major depressive disorder (MDD) is a mental disease characterized by pervasive and persistent low mood with loss of pleasure, feelings of guilt, and inferiority. The lifetime prevalence of depressive disorder among Chinese adults is 6.8%, with 3.4% for MDD (Lu et al., 2021). MDD is a multifactor disease with both environmental and genetic factors playing a role. A previous epidemiological study using a large patient cohort identified adverse life events, particularly in childhood, that were highly associated with the onset of MDD, with its effects persisting beyond childhood (Kessler et al., 1997). Furthermore, spousal violence has also been identified as a risk factor for MDD, with a twofold to threefold higher probability compared to non-exposed women (Beydoun et al., 2012). In addition to strong evidence of environmental factors contributing to the disease, genetic predisposition has also been identified as a factor of MDD. A previous meta-analysis demonstrated that the heritability of MDD was approximately 31–42% (Sullivan et al., 2000). This is much lower compared to other mental diseases, such as schizophrenia, which is estimated to be approximately 70% (Sullivan et al., 2003). The interaction of gene and the environment has drawn increasing attention. Life event such as having a stressful life have been highly correlated with MDD and are partly influenced by genetic factors (Kessler, 1997; Kendler et al., 1999). In addition to life events, individuals mistreated during childhood have a high susceptibility to develop MDD, which in turn has been associated with genetic and epigenetic factors (Teicher and Samson, 2013).

During the interaction between genes and environment, epigenetic factors may play a critical role in the pathogenesis of MDD. A previous study found that children who were abused had a site-specific methylation at NR3C1, suggesting the potential role of DNA methylation in the interaction of gene-environment (McGowan et al., 2009). A study on genome-wide cytosine methylation patterns in mice found differential methylation following exposure to chronic social defeat stress (CSDS) in susceptible animals (O’Toole et al., 2019). These studies suggested that DNA methylation could be used to evaluate and predict depression.

Recent studies have demonstrated the predictive power of DNA methylation biomarkers in aging (Bell et al., 2019) and cancer (Pan et al., 2018). Additionally, the role of DNA methylation in psychiatric disorders has been demonstrated in numerous studies. A recent study demonstrated that BDNF DNA methylation was related to depression and could be used as a blood biomarker for MDD (Fuchikami et al., 2011). A study of postpartum depression demonstrated that DNA methylation of HP1BP3 and TTC9B could be used as predictors for postpartum depression with ∼80% accuracy (Guintivano et al., 2014). Additionally, a recent DNA methylation study on depression established a methylation risk score to predict long-term depression with an area under curve (AUC) of 0.724 (Clark et al., 2020). Estimators of biological age based on predictable age-related patterns of DNA methylation, so-called “epigenetic clocks,” have shown promise for their ability to capture accelerated aging in patients with depression (Protsenko et al., 2021). The studies mentioned above all support the notion that DNA methylation could be a promising biomarker to help diagnose and treat depression.

The relationship between depression and risk of breast cancer remains controversial. Numerous studies have shown no significant relationship between depression and breast cancer (Hahn and Petitti, 1988; Reeves et al., 2018). However, some studies have found that patients with depression had a higher risk of developing breast cancer. A 13-year prospective study found that among female patients with MDD, the risk of developing breast cancer was higher (Gallo et al., 2000; Gross et al., 2010). Prospective study in Asia found that the risk of developing breast cancer was 4.078 times higher in individuals with depression compared to individuals who were not depressed. This strongly suggested that depression was a predictor of breast cancer risk (Yeh and Lee, 2016). Furthermore, a meta-analysis found that depression was highly correlated with cancer recurrence and mortality (Wang et al., 2020). Another study demonstrated the relationship between childhood maltreatment and breast cancer, which was potentially due to alterations in immune-related gene expression, particularly in the classical NF-κB-related proinflammatory signaling pathway. Interestingly, childhood maltreatment was a strong predictor of adult depression by interacting with immune dysregulation (Bower et al., 2020). Overall, these studies provide important insights into the relationship between depression and breast cancer. A review published concluded that the assessment of depression may affect the investigation of the relationship between depression and breast cancer (Possel et al., 2012), thus an objective laboratory examination may help to elucidate the latent association between depression and breast cancer.

In this study, we investigated the association of DNA methylation at the gene level with depression and proposed a methylation-derived depression index (mDI) to predict depression. We subsequently validated the index to predict the risk of breast cancer.

## Materials and Methods

### Data Source

DNA methylation data for depression was extracted from the Gene Expression Omnibus (GEO) with the accession number GSE128235. The data consisted of 324 depressed and 209 healthy participants of European ethnicity recruited from the Max Planck Institute of Psychiatry. Depressed individuals were diagnosed using the Diagnostic and Statistical Manual of Mental Disorder (DSM) IV criteria. The demographic information of this cohort is shown in Table 1. Methylation profiles were obtained using the Illumina HumanMethylation450 BeadChip (450K), of which the details have been previously described (Zannas et al., 2019).

**TABLE 1:** Demographic information of the three data sets.

	Discovery dataset		Validation dataset		Breast cancer dataset^$^	
	Case	Control	Case	Control	Case	Control
Total	324	209	98	96	235	424
Age (Mean, SD)	47.51, 13.63	48.15, 13.17	45.87, 9.54	45.7, 10.01	52.45, 7.42	53.23, 7.19
Gender (Female/Male)	180/144	125/84	73/25	71/25	233/2	340/84

*^$^The case and control number is for the last follow-up (2010), the age is for the baseline.*

### SIMPO Algorithm

A previous study demonstrated that the difference between methylation of the gene body and promoter were significantly associated with gene expression with a correlation coefficient of 0.67, suggesting it to be a promising predictor of gene expression (Li et al., 2019). Based on this, we had previously proposed an algorithm, Statistical difference of DNA Methylation between Promoter and Other body region (SIMPO), to evaluate the DNA methylation value at gene level (Quan et al., 2021). Based on the SIMPO algorithm, our group achieved promising results for DNA methylation biomarker identification of type 2 diabetes (Liang et al., 2021) and colon cancer (Quan et al., 2020).

The input data for the SIMPO algorithm are the DNA methylation values of probes in the gene promoter and other regions (including the gene body, 3′UTR, 5′UTR, and 1stExon). *T*-test was used in the SIMPO algorithm, and the degree of difference between probes in the gene promoter and other regions (SIMPO score) was used to characterize the DNA methylation for each gene:

$$S\,i\,m\,P\,o\,s\,c\,o\,r\,e=\frac{\bar{x}-\bar{y}}{S_{w}\,\sqrt{\left(1/m\right)+\left(1/n\right)}}∼t\,\left(m+n-2\right),$$

Where,

$$S_{w}^{2}=\frac{1}{m+n-2}\,\left[\left(m-1\right)\,S_{1}^{2}\,\left(n-1\right)\,S_{2}^{2}\right]$$

Herein, $\bar{x}$ is the average DNA methylation value of probes located in the promoter region, $\bar{y}$ is the average DNA methylation value of probes located in the other regions, *m* is the number of probes located in the promoter region, *n* is the number of probes that are located in the other regions, $S_{1}^{2}$ is the variance of DNA methylation values of probes located in the promoter region, $S_{2}^{2}$ is the variance of DNA methylation values of probes located in the other regions.

### Prediction Model for Depression

We subsampled 90% of the DNA methylation data for depression 300 times without replacement, compared the difference of gene SIMPO values between cases and controls based on *t*-test and selected the top 50 genes ranked by the *p*-value of *t*-test for each iteration. As a result, we obtained a candidate gene list sorted by the number of occurrence in the top 50 genes for each iteration. Based on the gene list, we introduced an index, the methylation-derived Depression Index (mDI), by using the top *K* genes in the candidate gene list. The *K* genes were divided into a “positive” subgroup whose average t-scores were higher than 0 and a “negative” subgroup whose average t-scores were lower than 0. The mDI was derived from the statistical method *t*-test:

$$m\,D\,I=1+\frac{\bar{x}-\bar{y}}{\sqrt{\frac{S_{x}^{2}}{n}+\frac{S_{y}^{2}}{m}}}$$

Herein, $\bar{x}$ is the average SIMPO value of genes in the positive gene set, $\bar{y}$ is the average SIMPO value of genes in the negative gene set, $S_{x}^{2}$ is the variance of SIMPO values of genes in the positive gene set, $S_{y}^{2}$ is the variance of SIMPO values of genes in the negative gene set, *n* is the number of genes in the positive gene set, and *m* is the number of genes in the negative gene set.

The number of genes *K* ranging from 10 to 500 was used for mDI calculation and the best *K* was selected where mDI was the most significantly associated with depression based on the Pearson correlation method (with the highest correlation coefficient).

### Validation of Methylation-Derived Depression Index

To validate the predictive power of the prediction model, we tested whether our mDI model could be used in an independent dataset. The dataset was extracted from GEO with the accession number GSE113725. It included 98 individuals with a self-reported history of depression and 96 individuals without a self-reported history of depression or diagnosed mental health problems. The methylation profiles were obtained using the Illumina Infinium HumanMethylation450 BeadChip. The demographic information of the data set is provided in Table 1.

The mDI was used in the validation dataset. To validate the predictive power of mDI, we calculated the correlation between mDI scores and phenotype using the Pearson correlation method and compared the difference in mDI scores between cases and controls using a *t*-test.

### Functional Analysis and Network Analysis

Two gene expression datasets for MDD were used for comparison with the genes for mDI. One dataset was from Jansen et al. (2016) which compared the difference of gene expression between 882 subjects with current MDD and 331 healthy controls using peripheral blood samples. The other dataset was from brain tissues published by Labonté et al. (2017), which included 26 MDD samples and 22 controls. Gene enrichment analysis was performed on the genes for mDI calculation using Gene2Func in the functional mapping and annotation of genetic associations (FUMA) software (Watanabe et al., 2017). First, tissue specificity was evaluated using the differentially expressed gene (DEG) sets in GTEx v8 by employing a hypergeometric test (P_Bonferroni_ < 0.05). Then, a hypergeometric test was used to assess whether our genes were overrepresented in the predefined gene sets derived from Reactome. The false discovery rate (FDR) was controlled using the Benjamini–Hochberg method (FDR < 0.05). Based on the genes enriched in the pathways, we constructed a protein-protein interaction (PPI) network using the STRING database (Szklarczyk et al., 2019) with a confidence cutoff of 0.4. We identified PPI network modules using Molecular Complex Detection (MCODE) (Bader and Hogue, 2003) plugged in Cytoscape 3.9.0 (Shannon et al., 2003). The network modules with a degree cutoff of 2, node score cutoff of 0.2, k-core of 2, and max depth of 100 were extracted.

### Methylation Data for Breast Cancer

Breast cancer sample data in our study were extracted from the EPIC-Italy cohort obtained from Gene Expression Omnibus (GEO) with accession number GSE51032. This cohort was established at the Human Genetics Foundation (HuGeF) in Turin, Italy and was a prospective study aimed at investigating the etiology of cancer and other chronic diseases. The investigators recruited 659 participants at baseline, and evaluated the participants every year for breast cancer. The sample information is shown in Table 1. The number of diagnosed breast cancer patients and cancer-free participants at each follow-up is shown in Supplementary Table 1. At the last follow-up (2010), 424 individuals remained cancer-free, and 235 were diagnosed with breast cancer. The average age of the participants was 53 years old at the baseline and 87% of the participants were female (Riboli, 2001).

Whole blood samples were collected from all participants at baseline, and genome-wide DNA methylation patterns were profiled using the Infinium HumanMethylation450 BeadChip array. The cell proportions of the whole blood were calculated using the R *minfi* package using the DNA methylation signature (Houseman et al., 2012; Aryee et al., 2014). This included the proportion of T cells, B cells, NK cells, lymphocytes, monocytes, granulocytes, CD4 cells, CD8 cells, and the calculated ratio of CD4–CD8, the ratio of granulocytes to lymphocytes (NLR), and the ratio of monocytes to lymphocytes (MLR).

### Construction of Prediction Model for Breast Cancer

A previous study demonstrated that immune-inflammatory cells are an essential component for cancer progression and play an important role in tumor microenvironment (Hanahan and Weinberg, 2011). A meta-analysis showed that a high neutrophil-to-lymphocyte ratio (NLR) was found to be related to worse overall survival (OS) and disease-free survival (DFS) in patients diagnosed with breast cancer, and had a significant effect on estrogen receptor (ER)-negative and human epidermal growth factor receptor-2 (HER2)-negative patients (Ethier et al., 2017). To increase the accuracy of our prediction model, we used the mDI scores and cell proportion data as the predictor variables and phenotype *y* as the response variable. It was defined as 1 if the study participants developed primary breast cancer and 0 if they were cancer-free. Considering that the cell proportions ranged from 0 to 1 and the mDI was a t-score from the *t*-test, which included both positive and negative values, we performed normalization for all the independent variables. The normalization was performed using *z*-transformation:

$$z=\frac{x-m}{S}$$

Herein, *x* is one independent variable, *m* is the average value of the independent variable, and *S* is the standard deviation. After this process, we could convert the *z* value into a normal distribution with an average of 0 and standard deviation of 1.

Using the stepwise regression method, we constructed a model to predict the risk of breast cancer. Stepwise regression is a systematic method where terms are added and removed from a linear or generalized linear model based on their statistical significance to explain the response variable. In our study, we applied the *stepwiseglm* function in MATLAB to run the prediction model with the selected variables. In addition, we used R package *flexplot* (Fife, 2021) to compare the explained variance of our constructed model and the model without mDI. Furthermore, we applied random forest to construct the prediction model to further investigate the predictive potential of mDI (Breiman, 2001).

## Results

### Methylation-Derived Depression Index Prediction Model

**FIGURE 1:**
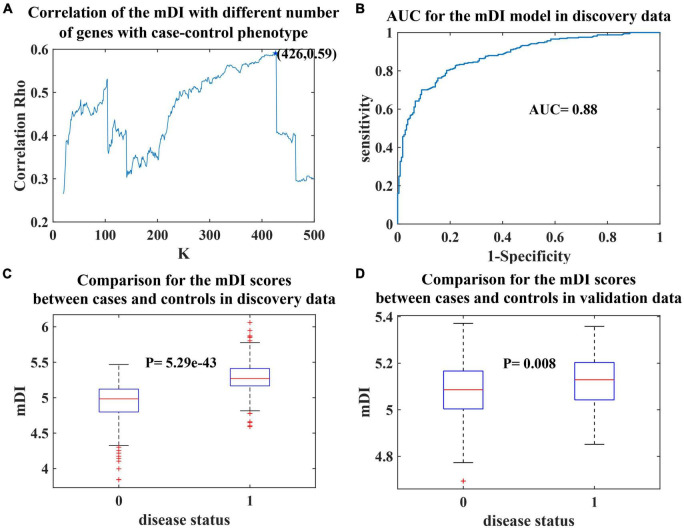
Prediction model for depression. **(A)** The number of genes (*K* = 10∼500) in the mDI (*x*-axis) plotted against the coefficient (*y*-axis). The curve plateaus at *K* = 426, with a coefficient of 0.59. **(B)** Receiver operating characteristic (ROC) curve of mDI. **(C)** Boxplot of mDIs for cases and controls in the discovery dataset. **(D)** Boxplot of mDIs for cases and controls in the validation dataset. The *p*-value derived from two-sample *t*-test.

We observed that the optimal mDI model was when the number of genes was 426, with coefficient = 0.59 and *p*-value = 2.06e-51 for the correlation between the mDI and case–control phenotype (Figure 1A). After that, the curve had a steep drop, and the correlation coefficient fluctuated at approximate 0.3. The gene list is provided in Supplementary Table 2 and consists of 245 “positive” genes and 181 “negative” genes. Based on the 426 genes, mDI was applied to the prediction model for depression. The receiver operating characteristic (ROC) curve for mDI had an area under the curve (AUC) of 0.88 (Figure 1B). We observed that the mDI of cases significantly differed from controls (*p*-value = 5.29e-43, Figure 1C), and a higher mDI indicates a strong risk of developing depression with an OR of 16.25. This suggested that mDI was a reliable model to classify depressed and healthy individuals.

### Validation of the Methylation-Derived Depression Index Prediction Model

To validate our prediction model, we used additional methylation dataset. A significant correlation between the mDI value and phenotypes was found in the validation dataset (coefficient = 0.19, *p*-value = 0.007). A significant difference in mDI scores between cases and controls was observed, with a *p*-value = 0.008 (Figure 1D). Furthermore, we observed a high risk of developing depression in the group with higher mDI scores, with an OR of 1.79 and the predictive power of mDI in the validation data generated an AUC of 0.60. These results validated our mDI model.

### Functional and Network Analysis of the Genes Used to Derive the Methylation-Derived Depression Index

**FIGURE 2:**
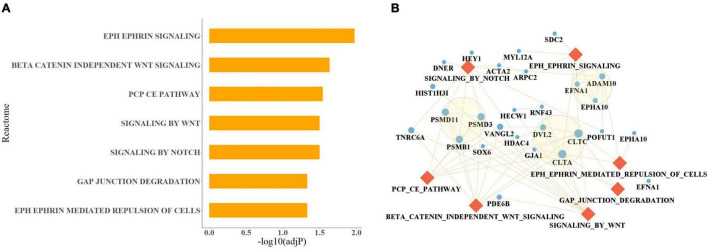
Pathway analysis results of the 426 genes selected in the mDI model. **(A)** Bar plot of enriched Reactome pathways that passed a Benjamini–Hochberg-adjusted *p*-value < 0.05. The length of the bar indicates the degree of significance. **(B)** Network of the enriched pathways and their involved genes, Gene interactions were extracted from STRING. Genes are drawn as blue circles where their size indicates the number of involved pathways, and pathways are drawn as orange diamonds. The interaction between pathways and involved genes is indicated by yellow lines, the interactions between genes are indicated by blue lines. The three modules identified by MCODE are highlighted with circles.

Among the 426 genes identified in our study, 128 genes were differentially expressed in MDD blood samples (Jansen et al., 2016) (FDR < 0.5), 103 and 94 genes were differentially expressed in female and male brain samples, respectively. Tissue-specific enrichment analysis showed that the 426 genes used in the mDI model showed significant enrichment in brain tissues, including putamen basal ganglia, amygdala, hippocampus, substantia nigra, anterior cingulate cortex BA24, caudate basal ganglia, frontal cortex BA9, nucleus accumbens basal ganglia, and hypothalamus (Supplementary Figure 1, Bonferroni corrected *p*-value < 0.05). In addition, pathway enrichment analysis of these genes revealed a total of 11 Reactome pathways. To achieve more specific enrichment, we excluded pathways with more than 500 genes. Seven pathways were enriched, included EPH Ephrin signaling, beta catenin-independent Wnt signaling, signaling by Wnt and signaling by Notch (Figure 2A).

Twenty five genes were found to be present in the seven enriched pathways. CLTC and CLTA were present in 6 of the pathways, PSMD11, PSMD3, and PSMB1 were present in four of the pathways, and ADAM10, VANGL2, TNRC6A, and DVL2 were present in three of the pathways (Figure 2B). The 25 genes were used to construct a protein–protein interaction (PPI) network (Figure 2B). In addition, three modules were identified. These modules included PSMB1, PSMD3, and PSMD11 that were associated with the proteasome; CLTA, CLTC, GJA1, and DVL2, which were associated with autophagy; and EPHA10, EFNA1, and ADAM10, which were involved in Ephrin signaling.

### Prediction Model for Breast Cancer

**FIGURE 3:**
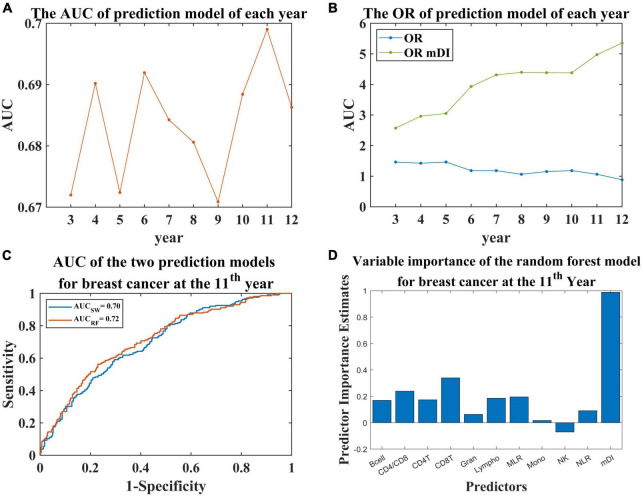
The results of the prediction model for breast cancer at various years. **(A)** AUC of the prediction models for each year. **(B)** OR of the prediction models. The blue line indicates the OR for the prediction model for each year; the green line indicates the OR for the comparison between the highest 25% of mDI scores and the lowest 25%. **(C)** The receiver operating characteristic (ROC) curve of the prediction model for breast cancer at the 11th year using two methods. AUC_sw_ indicates the AUC obtained by stepwise regression. AUC_RF_ indicates the AUC obtained by random forest. **(D)** Bar plot showing the predictive importance estimates of each predictor in the random forest prediction model.

Using mDI scores and cell proportion data as predictor variables and the diagnosis of breast cancer for each year as response variable, we constructed prediction models for breast cancer. Because the number of diagnosed individuals was limited during the first 2 years, we constructed the model using the data derived from the third year onwards. We observed the AUC curve for the prediction model for each year had a slightly increasing trend and then fluctuated at approximately 0.68 (Figure 3A). Furthermore, the ORs of the models for the different years were higher than 1 except for the last year (Figure 3B). To investigate the contribution of mDI to breast cancer, we divided the samples into 4 subsamples based on the fourth quantile of mDI scores and observed a higher risk in the highest 25% of mDI scores compared to the lowest 25% with ORs ranging from 2.57 to 5.35 (Figure 3B). For the prediction model at the 6th, 7th, 9th, 10th, and 11th years, the mDI interacted with the ratio of CD4 and CD8 to contribute to the prediction model (Supplementary Table 3).

The regression model at the 11th year is shown in Table 2, with an AUC of 0.70 (Figure 3C). For the regression models, we found that the *p*-value of mDI was significant (*p*-value < 0.05, Supplementary Table 3). Considering the potential bias introduced by age, we included age in the regression model, and found no significant contribution to the model (Supplementary Table 4). Furthermore, the correlation between mDI score and the ratio of CD4 and CD8 was significant, with coefficient = 0.558 and *p*-value = 0.048. To investigate the contribution of mDI in the model, we tried to remove mDI from the regression model at the 11th year, and found a significant change of R square (*P* = 0.004), implying the important contribution of mDI in our prediction model (Supplementary Table 5). Furthermore, we performed random forest to construct a prediction model at the 11th year. We observed a predictive model with as AUC of 0.72 (Figure 3C) with mDI having important contribution to the model (Figure 3D).

**TABLE 2:** Regression model results for the breast cancer prediction model at the 11th year.

Row	Estimate	SE	T Stat	*P*-value
**mDI**	**0.6037**	**0.1200**	**5.0324**	**4.843E-07**
Mono	1.1239	0.2997	3.7501	0.000177
Gran	4.4030	1.1066	3.9790	6.919E-05
Lympho	4.5001	1.1602	3.8787	0.000105
CD4/CD8	−0.4782	0.1767	−2.7066	0.006798
NLR	−0.2074	0.3227	−0.6427	0.520430
**mDI:CD4/CD8**	**0.5579**	**0.1976**	**2.8231**	**0.004756**
CD4/CD8:NLR	−0.3372	0.2014	−1.6744	0.094047

*The variables with P-value < 0.05 were marked as bold.*

## Discussion

In this study, we proposed a methylation-derived depression index (mDI) to predict depression. It was found to be highly related with depression, with a coefficient of 0.59 and AUC of 0.88 in the discovery dataset and a coefficient of 0.19 and AUC of 0.60 in the validation dataset. The mDI score was then used to construct a prediction model for breast cancer risk by combining blood cell proportion data. We observed high predictive power with mDI making important contribution to the overall reliability of the model.

DNA methylation is extensively involved in biological activities. Several studies have demonstrated that DNA methylation plays an important role in the nervous system (Martinowich et al., 2003; Moore et al., 2013). Emerging evidence has also shown that DNA methylation participates in the pathogenic mechanism of stress-related psychiatric disorders, such as MDD (Klengel et al., 2014). Using DNA methylation as a biomarker to predict psychiatric disorders has gradually gained attention in recent years. Kundakovic et al. found that DNA methylation of BDNF could be a predictor for early life adversity, and changes in DNA methylation in blood could be a predictor of changes in the brain (Kundakovic et al., 2015). In our study, we integrated the DNA methylation values of genes using the SIMPO algorithm, and then identified difference in SIMPO scores between patients and healthy controls. Using this approach, we obtained the associated genes based on DNA methylation. We then calculated mDI using these associated genes to predict depression. mDI was found to be a strong predictor, which was validated using an additional cohort. Our results demonstrated that DNA methylation was a latent biomarker to understand the underlying mechanism of MDD and was useful for diagnosis and treatment.

Of the 426 genes used to construct the prediction model for depression, several genes were previously known to be associated with depression. IGF1 was found to be the most significantly different between patients and controls (*p*-value = 1.38e-4). It functions in regulating body growth and development and has been demonstrated to play a role in MDD. A previous study found significantly higher levels of IGF1 in patients compared to healthy controls (Kopczak et al., 2015). To compare the relation between IGF1 and treatment response, the authors compared the levels of IGF1 in patients with a Hamilton depression rating scale (HAM-D) 21-item score < 10 after 6 weeks of psychopharmacological treatment and those without remission. They found that remitters had a lower level of IGF1 compared to non-remitters. In addition, knockout of the IGF1 gene induced depressive symptoms in mice (Mitschelen et al., 2011). These results demonstrated that IGF1 could be a potential risk factor for MDD.

Tissue enrichment analysis showed that the selected 426 genes were strongly expressed in several brain regions, such as the hippocampus, amygdala and frontal cortex. At present, there is no consensus regarding specific brain regions correlated with MDD pathogenesis, however, several depression symptoms have been related to the dysfunction of certain brain regions. The neocortex and hippocampus have been shown to regulate the cognitive aspects of MDD, the striatum and amygdala have been shown to be involved in emotional memory, and the hypothalamus has been shown to be associated with neurovegetative symptoms such as too much or too little sleep, energy and appetite (Nestler et al., 2002). There is a body of evidence showing that the frontal cortex plays a vital role in the development of depression, and has been considered as a treatment target (Hare and Duman, 2020). The genetic and chemical changes in these regions may provide new insight into the mechanism of depression.

Furthermore, we observed that the selected genes were significantly enriched in Eph Ephrin signaling, which is important for regulating the migration of neuronal cells and developmental plasticity of synapses (Kania and Klein, 2016). Increasing evidence has also demonstrated the relationship between inflammation and depression (Benton et al., 2007; Goldberg, 2010). In our study, immune-related pathways, such as beta catenin-independent Wnt signaling and Wnt signaling, were enriched. Wnt signaling has been correlated with neural development (Ille and Sommer, 2005) and found to play an important role in preventing postsynaptic damage induced by Abeta oligomers in hippocampal neurons (Cerpa et al., 2010). Several studies have also shown that the Wnt pathway to be an important mediator of MDD (Sani et al., 2012). Studies have shown increased expression of Wnt2 in rats after treatment with antidepressants (Okamoto et al., 2010). Notch signaling has also been shown to be associated with brain morphogenesis (Fischer-Zirnsak et al., 2019). Results from this study showed Notch signaling to be enriched, implying its potential role in the etiology of MDD.

We then constructed a PPI network using the selected genes involved in the enriched pathways. This generated three modules, of which, the first module comprised of PSMD11, PSMD3, and PSMB1, which encode important subunits of the proteasome. The proteasome is widely distributed in eukaryotic cells and serves as a proteolytic system that is dependent on ubiquitin. The ubiquitin-proteasome system (UPS) regulates neural development and maintains the structure and biological function of the brain. UPS has been found to be related to schizophrenia (Luza et al., 2020). A study that compared schizophrenia patients with healthy controls found that expression levels of genes encoding proteasome subunits and ubiquitin were reduced, suggesting that hypofunction of the UPS may contribute to schizophrenia (Altar et al., 2005). Based on our results, the role of the UPS in MDD needs to be further investigated. The second module comprised of CLTA, CLTC, GJA1, and DVL2. CLTC is an important gene related to autophagy (Latomanski and Newton, 2018). Neuroinflammation is an important mechanism related to MDD. A study found that lipopolysaccharide-induced depressive-like behavior impaired the autophagy system. Melatonin was found to significantly improve autophagy function, suggesting that melatonin may mediate autophagy through FOXO3a signaling (Ali et al., 2020). This provides evidence of the important function of these two major cellular quality control systems in psychiatric disorders and provides opportunities for targeted treatment of MDD. The third module comprised of ADAM10, EFNA1, and EPHA10. ADAM10 is a member of the ADAM family that participates in regulating cell adhesion, migration, and signaling. ADAM10 plays a major role in the Notch and Eph/ephrin pathways (Edwards et al., 2008). Studies have demonstrated that ADAM10 deficiency was linked to dysfunction of the central nervous system (Saftig and Lichtenthaler, 2015). These results suggest that ADAM10 may be a risk factor in MDD pathogenesis by targeting ephrin pathways.

Lastly, to investigate the value of mDI and validate its predictive power, we used it to predict the risk of breast cancer. The relationship between depression and breast cancer has been a topic of contention. However, several studies have found that patients with depression have a higher risk of breast cancer (Gallo et al., 2000; Gross et al., 2010). In this study, we combined mDI and cell proportion data to construct a prediction model for breast cancer. Our results demonstrated that the model was highly predictive of the risk of breast cancer. After removing mDI and its related interaction terms, we found a significant decrease in explained variance of the model. Furthermore, the random forest model justified the important contribution of mDI in the prediction model for breast cancer. Interestingly, the interaction of mDI and the ratio of CD4 and CD8 strongly contributed to the prediction model. We also found a significant correlation between mDI scores and the ratio of CD4 and CD8, suggesting an immune mechanism for depression.

There were several limitations to the present study. First, the sample size of the datasets we used may have not been sufficient to comprehensively detect all methylation markers related to depression. This may be the reasons for the low predictive power of the model in our validation dataset. The second limitation was information on confounding factors such as smoking and ethnicity, was not available, and hence may have contributed to bias in our model. Third, the DNA methylation profiles of whole blood samples may not reveal the complete mechanism of epigenetic effects on depression, especially in brain tissues. The comparison with gene expression data showed that the overlap between DNA methylation genes and differentially expressed genes from the different samples was limited. The current version of mDI included the DNA methylation data from 426 genes. This huge number may limit its potential in clinical applications. In this study, we primarily demonstrated the contribution of mDI to predict depression. We intent to analyze a larger cohort in the future and generate more comprehensive models by combining DNA methylation data with clinical biochemical results.

## Conclusion

In conclusion, we found that our methylation-derived depression index was highly associated with depression and had significant predictive power. Furthermore, our model could be used to predict the risk of breast cancer with significant reliability. Biological function analysis of the selected genes also provided clues for the mechanism of depression and provided insights into the role of DNA methylation in the pathogenesis of depression. This is valuable for the diagnosis and treatment of depression.

## Data Availability Statement

The original contributions presented in the study are included in the article/Supplementary Material, further inquiries can be directed to the corresponding author/s.

## Author Contributions

JX, TY, and DT conceived the study. FL and XH downloaded the data. NW and JS implemented the computational framework. TP revised the coding, analyzed the data, and drafted the manuscript. HZ contributed to the explanation of the result. SC revised the manuscript with input from all authors. SC and JX supervised the study and were in charge of overall direction and planning.

## Conflict of Interest

JX was a cofounder of Deepome Inc. The remaining authors declare that the research was conducted in the absence of any commercial or financial relationships that could be construed as a potential conflict of interest.

## Publisher’s Note

All claims expressed in this article are solely those of the authors and do not necessarily represent those of their affiliated organizations, or those of the publisher, the editors and the reviewers. Any product that may be evaluated in this article, or claim that may be made by its manufacturer, is not guaranteed or endorsed by the publisher.
