# Supplementary material for: DNA Methylation Markers and Prediction Model for Depression and Their Contribution for Breast Cancer Risk
Source: Front Mol Neurosci. 2022 Feb 23;15:845212. doi: 10.3389/fnmol.2022.845212 (PMC8904753; doi:10.3389/fnmol.2022.845212)

**Supplementary Information**

Table of contents

Table S1. The number of diagnosed breast cancer patients and cancer-free participants at each follow-up period………………………………………………………….….2

Table S2. Gene list for calculating mDI for depression prediction. T-test was used to estimate the difference of SIMPO value between patients and controls in the discovery dataset………………………………………………………………………3

Table S3. The parameters for the prediction model for breast cancer…………….13

Table S4. The parameters for the regression model after adding age…………….….16

Table S5. The difference of explained variance of the regression model after removing mDI ……………………………………………………………………...16

Figure S1. Tissue specificity of genes derived from gene2func (53 specific tissues) GTEx v8…………………………………………………………………………….. 17

| **Table S1.** The number of diagnosed breast cancer patients and cancer-free participants at each follow-up | | |  |
| --- | --- | --- | --- |
| year | diagnosed cases | cancer-free | |
| 1 | 23 | 636 | |
| 2 | 66 | 593 | |
| 3 | 85 | 574 | |
| 4 | 103 | 556 | |
| 5 | 120 | 539 | |
| 6 | 140 | 519 | |
| 7 | 155 | 504 | |
| 8 | 171 | 488 | |
| 9 | 184 | 475 | |
| 10 | 199 | 460 | |
| 11 | 215 | 444 | |
| 12 | 235 | 424 | |

| **Table S2.** Gene list for calculating mDI for depression prediction. T-test was used to estimate the difference of SIMPO value between patients and controls in the discovery dataset. | | | |
| --- | --- | --- | --- |
| **Gene** | **T-test score** | **T-test-p-value** | **Direction** |
| IGF1 | 3.84532726 | 0.000137707 | pos |
| NUDT16 | 3.20332195 | 0.001441859 | pos |
| SLC38A6 | 3.19074967 | 0.001503329 | pos |
| KCNG2 | 3.07118417 | 0.002248801 | pos |
| MARCH8 | 3.09238106 | 0.002105218 | pos |
| TMEM126B | 3.01791587 | 0.002697984 | pos |
| PDS5B | 3.02789209 | 0.002614731 | pos |
| SLC15A3 | 3.04437576 | 0.002473224 | pos |
| KRT78 | 2.93542587 | 0.003516808 | pos |
| RNF43 | 2.98343482 | 0.00300496 | pos |
| VANGL2 | 2.95615584 | 0.003273592 | pos |
| SPAG17 | 2.99566212 | 0.002901972 | pos |
| FITM1 | 2.95662302 | 0.003282068 | pos |
| CRKL | 2.93709827 | 0.003483642 | pos |
| SKIL | 2.90615753 | 0.003836554 | pos |
| TMEM52 | 2.92525652 | 0.003639434 | pos |
| THRB | 2.8751699 | 0.004262641 | pos |
| ACER2 | 2.80866212 | 0.005209652 | pos |
| NEU2 | 2.82566415 | 0.004953867 | pos |
| TYK2 | 2.86234599 | 0.004399939 | pos |
| HECW1 | 2.80822351 | 0.005224412 | pos |
| SH3BP2 | 2.82513135 | 0.004953287 | pos |
| TNPO1 | 2.76529429 | 0.005922193 | pos |
| WNK2 | 2.77134844 | 0.005838821 | pos |
| IRAK2 | 2.77487226 | 0.005762756 | pos |
| PSMD11 | 2.71421832 | 0.006897224 | pos |
| RPL39L | 2.7189103 | 0.006805484 | pos |
| FAM3C | 2.70442919 | 0.007126162 | pos |
| KIAA1644 | 2.67338026 | 0.007808449 | pos |
| MTMR11 | 2.666883 | 0.007948234 | pos |
| ZNF805 | 2.70190341 | 0.007267204 | pos |
| HOXD9 | 2.61614485 | 0.009172684 | pos |
| PROCA1 | 2.68125157 | 0.007658847 | pos |
| TRIM35 | 2.61766806 | 0.009122948 | pos |
| RNF165 | 2.59614531 | 0.009716391 | pos |
| SUPT7L | 2.5994028 | 0.009636971 | pos |
| IMMP2L | 2.59480035 | 0.009801879 | pos |
| KCNC1 | 2.59025149 | 0.009894672 | pos |
| N4BP2L2 | 2.56505732 | 0.010640488 | pos |
| MCAT | 2.55290816 | 0.011009102 | pos |
| BHLHE41 | 2.60943225 | 0.009488462 | pos |
| TET2 | 2.54937107 | 0.011147183 | pos |
| TRIP4 | 2.62983773 | 0.008833015 | pos |
| DNER | 2.56640802 | 0.010590238 | pos |
| TMEM67 | 2.53274891 | 0.011708198 | pos |
| ATP6V0B | 2.54476294 | 0.011294361 | pos |
| GPR182 | 2.53209126 | 0.011641186 | pos |
| FAM65B | 2.47639627 | 0.013631643 | pos |
| ADAM10 | 2.35657867 | 0.018850041 | pos |
| TBKBP1 | 2.542221 | 0.011372264 | pos |
| SFRS5 | 2.4040351 | 0.016581576 | pos |
| CDC25C | 2.47016454 | 0.013885692 | pos |
| PIGB | 2.45468831 | 0.014462293 | pos |
| STX1B | 2.46200262 | 0.014168842 | pos |
| SNRPA1 | 2.37770419 | 0.017838562 | pos |
| TRIM4 | 2.45596879 | 0.014465761 | pos |
| ANKS3 | 2.40503592 | 0.016574636 | pos |
| BAIAP2 | 2.44713568 | 0.014786593 | pos |
| SLC25A42 | 2.41889398 | 0.015939828 | pos |
| DAPK3 | 2.41948 | 0.015912453 | pos |
| SENP5 | 2.43369518 | 0.015353008 | pos |
| TGM3 | 2.35138619 | 0.019150804 | pos |
| BNIP3L | 2.40098275 | 0.016734468 | pos |
| PTDSS2 | 2.32974898 | 0.020276588 | pos |
| HOXC10 | 2.41421489 | 0.016200087 | pos |
| TMEM143 | 2.37919411 | 0.017846053 | pos |
| A2LD1 | 2.37802878 | 0.017849022 | pos |
| STIM1 | 2.35336956 | 0.019009448 | pos |
| GALNS | 2.4059234 | 0.016531186 | pos |
| GPR179 | 2.43296004 | 0.015348308 | pos |
| MAPK15 | 2.38310367 | 0.017573894 | pos |
| RNF216L | 2.33629181 | 0.019876162 | pos |
| RTDR1 | 2.40318684 | 0.016658385 | pos |
| TNRC6A | 2.4447783 | 0.014901225 | pos |
| C21orf57 | 2.35908979 | 0.01878247 | pos |
| OBFC1 | 2.4189811 | 0.015963128 | pos |
| ZBTB11 | 2.29593274 | 0.022134842 | pos |
| C5orf22 | 2.13316954 | 0.033423162 | pos |
| HLA-DPB2 | 2.29348621 | 0.022291381 | pos |
| LTBP2 | 2.3607161 | 0.018660599 | pos |
| C20orf196 | 2.31189307 | 0.021256744 | pos |
| HELZ | 2.3723617 | 0.018172438 | pos |
| LCE6A | 2.25080115 | 0.024902358 | pos |
| HIST1H3I | 2.29123211 | 0.022439313 | pos |
| KRT4 | 2.3118084 | 0.021225789 | pos |
| TUSC2 | 2.35596952 | 0.018871855 | pos |
| DPYD | 2.21471742 | 0.027298319 | pos |
| HEY1 | 2.23903806 | 0.02569559 | pos |
| COL8A1 | 2.34119486 | 0.019656445 | pos |
| MRGPRE | 2.20858635 | 0.027747449 | pos |
| PSMB1 | 2.23811127 | 0.025658064 | pos |
| INSL6 | 2.1747524 | 0.030184405 | pos |
| SCNN1G | 2.26651112 | 0.02394217 | pos |
| ACTR1A | 2.2328323 | 0.026028434 | pos |
| CAB39L | 2.27963411 | 0.023082984 | pos |
| E2F8 | 2.25324059 | 0.024783571 | pos |
| MYH7 | 2.2024391 | 0.028150601 | pos |
| SLC22A16 | 2.32712405 | 0.020426803 | pos |
| ABCB10 | 2.35201295 | 0.019226099 | pos |
| C13orf39 | 2.23568896 | 0.025844595 | pos |
| DVL2 | 2.2852478 | 0.022806946 | pos |
| FAM190B | 2.20853779 | 0.027742685 | pos |
| HDAC4 | 2.1925198 | 0.028870604 | pos |
| KRT26 | 2.08151984 | 0.037894182 | pos |
| RASGRP3 | 2.16981496 | 0.030494495 | pos |
| SEC23B | 2.19770587 | 0.028487687 | pos |
| TNFRSF6B | 2.18202083 | 0.029592526 | pos |
| CLTC | 2.26058495 | 0.02433654 | pos |
| EFNA1 | 2.17486027 | 0.030187292 | pos |
| LRP1 | 2.28858956 | 0.022570398 | pos |
| N4BP3 | 2.20507208 | 0.027946933 | pos |
| SPERT | 2.19941949 | 0.028310246 | pos |
| FAM162B | 2.21446149 | 0.027346843 | pos |
| USP2 | 2.25449285 | 0.024700081 | pos |
| ALPPL2 | 2.21200058 | 0.027461654 | pos |
| C6orf222 | 2.15305555 | 0.031859395 | pos |
| C6orf94 | 2.1668425 | 0.03074503 | pos |
| CLN6 | 2.19899472 | 0.028378736 | pos |
| CPSF7 | 2.25155937 | 0.024806246 | pos |
| FASTK | 2.21986292 | 0.026913434 | pos |
| GJA1 | 2.09922814 | 0.036409775 | pos |
| MRPL28 | 2.19022982 | 0.029084354 | pos |
| SOX6 | 2.21483016 | 0.027318201 | pos |
| SUSD5 | 1.98296274 | 0.048108226 | pos |
| ZSCAN21 | 2.25688316 | 0.024628077 | pos |
| DEPDC6 | 2.1281942 | 0.033910484 | pos |
| METTL10 | 2.07015066 | 0.03906562 | pos |
| TTC22 | 2.14813377 | 0.032315763 | pos |
| ALDH3B1 | 2.09297004 | 0.036928472 | pos |
| ARHGAP27 | 2.14595622 | 0.032447316 | pos |
| LRRC16A | 2.09329061 | 0.036878978 | pos |
| MED26 | 2.1327427 | 0.033467751 | pos |
| MT1E | 2.13220467 | 0.033545519 | pos |
| PFDN4 | 2.25280418 | 0.02478276 | pos |
| PGAM2 | 2.03638699 | 0.042310127 | pos |
| THOC5 | 2.13262756 | 0.033462414 | pos |
| TRIM5 | 2.15155446 | 0.031955122 | pos |
| AP4E1 | 2.13185872 | 0.033566531 | pos |
| ARPC2 | 2.08223455 | 0.037874333 | pos |
| DCUN1D2 | 2.02881857 | 0.043087141 | pos |
| FLJ39653 | 2.13458048 | 0.033274865 | pos |
| FLT3 | 2.09418474 | 0.036785777 | pos |
| GOLSYN | 2.18958106 | 0.02908617 | pos |
| HDGF | 2.03347571 | 0.042576236 | pos |
| KIAA1191 | 2.02600988 | 0.043332246 | pos |
| LOC283174 | 2.14976882 | 0.032160346 | pos |
| LPPR5 | 2.22093442 | 0.026908813 | pos |
| LRRC66 | 2.05867327 | 0.040055021 | pos |
| PCBD2 | 2.12034379 | 0.034522073 | pos |
| PKNOX2 | 2.02856179 | 0.043165368 | pos |
| PRPF38B | 2.07621116 | 0.038372533 | pos |
| RAPGEF3 | 2.06008936 | 0.03998379 | pos |
| SERPINA6 | 2.01498187 | 0.044491537 | pos |
| SPINLW1 | 2.08241614 | 0.037886154 | pos |
| ST3GAL1 | 2.06747409 | 0.039299828 | pos |
| SYCE1L | 2.06801121 | 0.039245916 | pos |
| TCF19 | 2.02876107 | 0.043097449 | pos |
| WDR5 | 2.16113235 | 0.031203411 | pos |
| ALS2CR4 | 1.99380041 | 0.046850456 | pos |
| ATAD2 | 1.83301749 | 0.067507189 | pos |
| C10orf2 | 2.11003112 | 0.03551084 | pos |
| C2orf3 | 2.13019758 | 0.033732615 | pos |
| DNAJC24 | 1.51250372 | 0.13115828 | pos |
| FAM132A | 2.0711339 | 0.038883826 | pos |
| GDE1 | 2.0717807 | 0.038789223 | pos |
| GPER | 1.953337 | 0.051411544 | pos |
| IER5L | 2.07669883 | 0.038392894 | pos |
| IMMT | 2.03222801 | 0.042722768 | pos |
| IQGAP1 | 2.12695258 | 0.033963753 | pos |
| ISM2 | 2.06682255 | 0.039386411 | pos |
| ITPRIP | 1.9148799 | 0.056224236 | pos |
| KCND3 | 2.07235433 | 0.038872395 | pos |
| LCN8 | 2.07920346 | 0.03812483 | pos |
| LOC283392 | 1.99599331 | 0.046493724 | pos |
| LOC285696 | 1.94243777 | 0.052743928 | pos |
| LRGUK | 2.02977633 | 0.042985438 | pos |
| NEK6 | 1.9919687 | 0.046951916 | pos |
| PIWIL2 | 2.05099497 | 0.040902358 | pos |
| PKIG | 1.91098896 | 0.056621313 | pos |
| PLD2 | 1.9727801 | 0.049172911 | pos |
| QSOX1 | 2.06741122 | 0.03926151 | pos |
| SAAL1 | 1.97364544 | 0.049057095 | pos |
| TMEM72 | 2.12560017 | 0.034118284 | pos |
| TUBG2 | 2.04686769 | 0.04128733 | pos |
| TUG1 | 1.96867773 | 0.049637833 | pos |
| ZNF256 | 2.08864306 | 0.037229725 | pos |
| ZNF346 | 2.07277133 | 0.038705862 | pos |
| ZNHIT1 | 2.00427074 | 0.04570996 | pos |
| ACSS1 | 2.08131458 | 0.038031459 | pos |
| ATG4D | 1.89731528 | 0.058463267 | pos |
| BDKRB2 | 2.02942005 | 0.042997643 | pos |
| C10orf41 | 2.08128655 | 0.037966668 | pos |
| CCDC130 | 2.00702246 | 0.045412802 | pos |
| CD37 | 2.06187535 | 0.039811516 | pos |
| CLDN1 | 2.15040534 | 0.03205743 | pos |
| CLDN12 | 1.84455015 | 0.065841938 | pos |
| CRYBA2 | 2.03855855 | 0.04215442 | pos |
| CTSC | 1.99438117 | 0.046718413 | pos |
| CYP1B1 | 1.84810114 | 0.065253054 | pos |
| CYP26A1 | 2.06353887 | 0.039638133 | pos |
| D2HGDH | 1.87889374 | 0.060894003 | pos |
| DENND2A | 1.98215743 | 0.048002122 | pos |
| EPHA10 | 1.87384779 | 0.061699093 | pos |
| FAM193A | 1.9835585 | 0.047905269 | pos |
| FGD2 | 2.02430544 | 0.043542627 | pos |
| HAMP | 2.17576509 | 0.030113067 | pos |
| LYSMD4 | 2.0280024 | 0.043164453 | pos |
| MANEAL | 1.9655655 | 0.050003597 | pos |
| MARK3 | 2.09241562 | 0.037029069 | pos |
| MN1 | 2.09560688 | 0.036626624 | pos |
| NADK | 2.04465849 | 0.041426389 | pos |
| NINJ2 | 2.05690138 | 0.040255969 | pos |
| NOLC1 | 1.80765772 | 0.071330376 | pos |
| OOEP | 2.13411346 | 0.033341638 | pos |
| PARVA | 2.15281655 | 0.031912516 | pos |
| PCDHB19P | 1.9289663 | 0.054301908 | pos |
| PCDP1 | 2.14225993 | 0.032671823 | pos |
| PDGFD | 1.95871491 | 0.050823263 | pos |
| POFUT1 | 2.12083906 | 0.034482598 | pos |
| POGZ | 1.74866661 | 0.081046704 | pos |
| PSMD3 | 1.80067922 | 0.072464326 | pos |
| RALGAPA2 | 1.80614969 | 0.071563781 | pos |
| RFTN1 | 2.05269501 | 0.040677104 | pos |
| SEC24A | 1.92632222 | 0.054668595 | pos |
| SNX25 | 1.94834316 | 0.05201911 | pos |
| SPARC | 1.91139767 | 0.056623715 | pos |
| SPO11 | 2.12755906 | 0.033962887 | pos |
| TACSTD2 | 1.94356871 | 0.052539295 | pos |
| TCEB1 | 1.9667632 | 0.049782549 | pos |
| TEKT3 | 2.14388043 | 0.032582486 | pos |
| TMEM45A | 1.94504634 | 0.05236628 | pos |
| TSPAN10 | 1.91530017 | 0.056102687 | pos |
| XKR4 | 2.09577922 | 0.036739723 | pos |
| YWHAQ | 2.03881584 | 0.04201568 | pos |
| ZDHHC3 | 1.9675698 | 0.049682924 | pos |
| ZFP37 | 2.07692673 | 0.038393862 | pos |
| ABP1 | 1.85987946 | 0.063546223 | pos |
| ACTA2 | 2.05332611 | 0.040652468 | pos |
| AGPHD1 | 1.85675752 | 0.063986909 | pos |
| ALDH5A1 | 2.00119522 | 0.045977637 | pos |
| ASAP3 | 2.00982198 | 0.045086862 | pos |
| ATAD3A | 1.67114589 | 0.095464931 | pos |
| BBOX1 | 1.98775244 | 0.047457949 | pos |
| BCL2L15 | 1.90791171 | 0.0570614 | pos |
| BSCL2 | 1.97130419 | 0.049337206 | pos |
| PDE6B | -4.155622 | 3.89296E-05 | neg |
| BBS7 | -3.6429298 | 0.000299609 | neg |
| COX16 | -3.4970603 | 0.000523281 | neg |
| PPP1R13B | -3.4759476 | 0.000556004 | neg |
| GALNTL2 | -3.1753381 | 0.001611743 | neg |
| GSDMB | -3.2421696 | 0.001268683 | neg |
| FANCL | -3.1373608 | 0.001828304 | neg |
| CADM3 | -3.0004153 | 0.002851414 | neg |
| GMFG | -2.9616841 | 0.00325753 | neg |
| TAS1R2 | -2.9786127 | 0.00306681 | neg |
| ORC4L | -2.9544087 | 0.003275673 | neg |
| DLC1 | -2.9838107 | 0.003015363 | neg |
| ZNF480 | -2.9447007 | 0.00342395 | neg |
| PIGR | -2.9131914 | 0.0037475 | neg |
| TTC21B | -2.9042573 | 0.003838044 | neg |
| VCAN | -2.807832 | 0.005171134 | neg |
| PLD6 | -2.7770327 | 0.005752482 | neg |
| GUCA2A | -2.7937868 | 0.005438826 | neg |
| RASL10A | -2.8110822 | 0.005178883 | neg |
| GABRB1 | -2.8288987 | 0.004890025 | neg |
| HGC6.3 | -2.7781926 | 0.005698328 | neg |
| TDH | -2.753849 | 0.006127656 | neg |
| SBK2 | -2.7388195 | 0.006401347 | neg |
| SDC2 | -2.7505749 | 0.006178908 | neg |
| LMO3 | -2.7191577 | 0.006789693 | neg |
| GLG1 | -2.7125903 | 0.006907462 | neg |
| ITPRIPL2 | -2.7197518 | 0.006789763 | neg |
| STK11IP | -2.7294221 | 0.006593533 | neg |
| RBM6 | -2.691995 | 0.00735067 | neg |
| ZNF193 | -2.6269444 | 0.008898475 | neg |
| TSKU | -2.6002366 | 0.009655133 | neg |
| DAO | -2.5801847 | 0.010164863 | neg |
| KLHL1 | -2.6245149 | 0.008992224 | neg |
| WDR67 | -2.6094021 | 0.0093377 | neg |
| FBXO17 | -2.5996828 | 0.009618406 | neg |
| DCAF15 | -2.5421578 | 0.01137905 | neg |
| LRRC17 | -2.5206613 | 0.012040913 | neg |
| MTO1 | -2.4760221 | 0.01361013 | neg |
| IL27RA | -2.5335812 | 0.011635868 | neg |
| ZNF528 | -2.4810694 | 0.013474188 | neg |
| TMC5 | -2.4894025 | 0.013220197 | neg |
| FAM119A | -2.5381645 | 0.011522245 | neg |
| FAM73A | -2.4554314 | 0.014439601 | neg |
| ZNF706 | -2.5079997 | 0.01246875 | neg |
| LRRC15 | -2.4619057 | 0.014228295 | neg |
| ASZ1 | -2.2500104 | 0.025038998 | neg |
| ZNF321 | -2.4048651 | 0.016586258 | neg |
| CD8B | -2.4249046 | 0.015677268 | neg |
| DNAL4 | -2.4060312 | 0.016519196 | neg |
| TMEM63B | -2.3815297 | 0.017686398 | neg |
| ENO2 | -2.4185558 | 0.016070103 | neg |
| TMEM185B | -2.4158742 | 0.016134095 | neg |
| AACSL | -2.4972297 | 0.012994004 | neg |
| FLNB | -2.439879 | 0.015064597 | neg |
| MND1 | -2.444236 | 0.014873666 | neg |
| MPZ | -2.3180895 | 0.020824236 | neg |
| SSB | -2.3249658 | 0.020485579 | neg |
| ING3 | -2.334832 | 0.020046981 | neg |
| GCN1L1 | -2.3251632 | 0.020508387 | neg |
| TIMM13 | -2.3216418 | 0.020682908 | neg |
| MTA2 | -2.3364328 | 0.019846959 | neg |
| SNF8 | -2.2948066 | 0.022194883 | neg |
| F13A1 | -2.3782748 | 0.01781856 | neg |
| CBL | -2.362889 | 0.01859048 | neg |
| WDR55 | -2.3968561 | 0.017207401 | neg |
| ATP6V0E1 | -2.3680823 | 0.018249053 | neg |
| GAB4 | -2.3342768 | 0.020042191 | neg |
| ME2 | -2.3322215 | 0.020088804 | neg |
| NLRP8 | -2.3714938 | 0.018142706 | neg |
| PNRC1 | -2.3321568 | 0.020138982 | neg |
| COX18 | -2.236943 | 0.025762212 | neg |
| CWF19L2 | -2.2505878 | 0.024845078 | neg |
| FAM195B | -2.3224287 | 0.020643419 | neg |
| PITX2 | -2.2398122 | 0.02559238 | neg |
| SNAPC5 | -2.1075116 | 0.035668182 | neg |
| C12orf10 | -2.2962948 | 0.022145451 | neg |
| IFIT5 | -2.2243624 | 0.026643884 | neg |
| ACY1 | -2.275969 | 0.023400805 | neg |
| LOC400931 | -2.3051679 | 0.021603926 | neg |
| NNT | -2.2966356 | 0.022110893 | neg |
| SMAD9 | -2.2520712 | 0.024852074 | neg |
| TKT | -2.2386443 | 0.025678942 | neg |
| HOOK2 | -2.3521514 | 0.019131282 | neg |
| GJA3 | -2.2213649 | 0.026841933 | neg |
| TGM4 | -2.2765824 | 0.023303642 | neg |
| TLX2 | -2.2689553 | 0.023747961 | neg |
| UHRF1BP1L | -2.272378 | 0.023539813 | neg |
| ALDH2 | -2.2966694 | 0.022084584 | neg |
| C20orf112 | -2.1952228 | 0.028652875 | neg |
| FOXN2 | -2.1571599 | 0.031557041 | neg |
| GLRA3 | -2.2025007 | 0.028155431 | neg |
| SOX8 | -2.1723527 | 0.030385625 | neg |
| NAB1 | -2.1712806 | 0.030378046 | neg |
| CD38 | -2.2960625 | 0.022164761 | neg |
| CFD | -2.2805973 | 0.023045539 | neg |
| CHRD | -2.1966648 | 0.028582414 | neg |
| CSF2 | -2.0126582 | 0.044743642 | neg |
| CSTF3 | -2.2576759 | 0.024592976 | neg |
| EIF2AK3 | -2.0906785 | 0.037045511 | neg |
| GPATCH2 | -2.2376056 | 0.025789992 | neg |
| NIPSNAP3A | -2.0789761 | 0.038245813 | neg |
| SLC30A4 | -1.8870308 | 0.059764938 | neg |
| TCF21 | -2.113533 | 0.035121578 | neg |
| C16orf52 | -2.1733984 | 0.030241287 | neg |
| CRYGN | -2.2793276 | 0.023109171 | neg |
| GPR37L1 | -2.192774 | 0.028853669 | neg |
| LRRC32 | -2.1707792 | 0.030555058 | neg |
| RGS10 | -2.2114436 | 0.027486308 | neg |
| TROAP | -2.28096 | 0.023018262 | neg |
| WBP2 | -2.2342838 | 0.026028718 | neg |
| ZBTB44 | -2.1906173 | 0.028967952 | neg |
| C6orf129 | -2.2040012 | 0.028022501 | neg |
| C6orf208 | -2.1286322 | 0.033857445 | neg |
| EDNRA | -2.1672728 | 0.03075909 | neg |
| POP5 | -2.0015813 | 0.045947727 | neg |
| ACTC1 | -2.2130293 | 0.027438522 | neg |
| C1orf172 | -1.9891963 | 0.047251183 | neg |
| C3 | -2.0331105 | 0.042639735 | neg |
| C4orf3 | -2.278216 | 0.023289577 | neg |
| DPP9 | -2.0519576 | 0.040769197 | neg |
| DYNLRB2 | -2.2646268 | 0.023942427 | neg |
| ETV1 | -2.1312054 | 0.033617471 | neg |
| HCG27 | -2.0039474 | 0.045658177 | neg |
| HN1 | -2.1911931 | 0.028993775 | neg |
| KCNJ6 | -2.170509 | 0.030523181 | neg |
| NR2C2AP | -2.1475544 | 0.032330044 | neg |
| PITPNA | -2.0818208 | 0.038006522 | neg |
| RPS23 | -2.0420463 | 0.041709777 | neg |
| SLC6A11 | -2.2086509 | 0.02779729 | neg |
| TMEM100 | -2.0967082 | 0.036556306 | neg |
| TMEM90A | -2.1326453 | 0.0334958 | neg |
| ALDH7A1 | -1.8980287 | 0.058275725 | neg |
| ARL5B | -2.1301471 | 0.033681145 | neg |
| BIRC6 | -2.0369288 | 0.042272301 | neg |
| C22orf34 | -2.1142751 | 0.03508517 | neg |
| C2orf68 | -1.9766945 | 0.048753212 | neg |
| F7 | -1.9094396 | 0.056901477 | neg |
| GPR144 | -2.0843927 | 0.037726109 | neg |
| HMGXB3 | -2.0202042 | 0.044064903 | neg |
| HSD11B2 | -1.7302206 | 0.084191226 | neg |
| HSD17B8 | -1.9723126 | 0.049167318 | neg |
| KHDC1 | -1.8496305 | 0.064997687 | neg |
| MYL12A | -2.1045121 | 0.035897788 | neg |
| PPP2R2B | -1.9632962 | 0.050256426 | neg |
| RCAN2 | -2.1893417 | 0.029050553 | neg |
| SIDT2 | -1.9037957 | 0.057531867 | neg |
| SYT2 | -2.0240819 | 0.043581119 | neg |
| TPRA1 | -1.877233 | 0.061102778 | neg |
| UPK1B | -2.0175988 | 0.0442019 | neg |
| BEGAIN | -2.0483145 | 0.041096235 | neg |
| BLNK | -1.8569461 | 0.06399348 | neg |
| C1QA | -1.9525879 | 0.051454 | neg |
| C1orf226 | -1.6173096 | 0.106471412 | neg |
| CCDC134 | -1.879298 | 0.06082944 | neg |
| CLTA | -1.8300613 | 0.067861914 | neg |
| ERCC1 | -1.9139753 | 0.056256252 | neg |
| GRIN2A | -1.8848532 | 0.060102458 | neg |
| GUCA2B | -1.9969862 | 0.046488172 | neg |
| HDHD2 | -2.0231963 | 0.043638667 | neg |
| HNRPLL | -1.9239698 | 0.054968725 | neg |
| MAP2K1 | -1.9184142 | 0.055690611 | neg |
| MKRN2 | -1.9675841 | 0.049692529 | neg |
| NAP1L5 | -1.9453317 | 0.052392168 | neg |
| NSUN7 | -1.7861767 | 0.074741866 | neg |
| PL-5283 | -1.9299562 | 0.054275962 | neg |
| PRX | -1.9934143 | 0.046809081 | neg |
| RNF122 | -2.0129011 | 0.044681279 | neg |
| SGMS2 | -2.0279467 | 0.043222161 | neg |
| SUSD1 | -2.0642509 | 0.039581901 | neg |
| TCF25 | -1.9964641 | 0.046537967 | neg |
| TFCP2 | -2.285283 | 0.022691909 | neg |
| TGM6 | -1.9907479 | 0.047150855 | neg |
| TRIM61 | -1.8814656 | 0.06052238 | neg |
| UBE2E1 | -1.9162545 | 0.056068269 | neg |
| VSIG2 | -1.8550027 | 0.064338291 | neg |
| ZNF677 | -1.9187638 | 0.055621272 | neg |
| AK3L1 | -2.0546931 | 0.040531068 | neg |
| ALPI | -1.8824888 | 0.060453934 | neg |
| APOB | -1.9153353 | 0.05607582 | neg |
| ATP5G2 | -1.9884944 | 0.047427543 | neg |
| BTD | -2.036676 | 0.04237375 | neg |

| **Table S3 Parameters used in the prediction model for breast cancer.** | | | | | | |  | | |  | | |  |
| --- | --- | --- | --- | --- | --- | --- | --- | --- | --- | --- | --- | --- | --- |
|  | **Row** | **Estimate** | | **SE** | | **tStat** | | | **pValue** | | |  |  |
|  | The model based on data in the 3^rd^ year | | | | | | | | | | | | |
|  | (Intercept) | | -2.14967 | | 0.146882 | | | -14.6353 | | | 1.67E-48 | | |
|  | mDI | | 0.269847 | | 0.135962 | | | 1.984717 | | | **0.047176** | | |
|  | CD8T | | -0.28022 | | 0.208823 | | | -1.34191 | | | 0.179626 | | |
|  | Bcell | | 0.039397 | | 0.155779 | | | 0.252906 | | | 0.800341 | | |
|  | Mono | | 0.936768 | | 0.42794 | | | 2.189017 | | | 0.028596 | | |
|  | Gran | | 3.275422 | | 1.591838 | | | 2.057635 | | | 0.039625 | | |
|  | Lympho | | 3.841954 | | 1.664364 | | | 2.308361 | | | 0.020979 | | |
|  | CD4/CD8 | | -0.71096 | | 0.313121 | | | -2.27056 | | | 0.023174 | | |
|  | CD8T:Mono | | 0.228993 | | 0.111037 | | | 2.062322 | | | 0.039177 | | |
|  | Bcell:CD4/CD8 | | 0.664635 | | 0.21619 | | | 3.074306 | | | 0.00211 | | |
| The model based on data in the 4^th^ year | | | | | | | | | | | | |  |
|  | (Intercept) | | -1.86232 | | 0.127453 | | | -14.6118 | | | 2.36E-48 | | |
|  | mDI | | 0.290052 | | 0.124016 | | | 2.338832 | | | **0.019344** | | |
|  | CD8T | | -0.23692 | | 0.192652 | | | -1.22981 | | | 0.21877 | | |
|  | Bcell | | 0.037464 | | 0.143124 | | | 0.261762 | | | 0.793505 | | |
|  | Mono | | 0.638185 | | 0.431704 | | | 1.478292 | | | 0.13933 | | |
|  | Gran | | 3.741062 | | 1.480378 | | | 2.527099 | | | 0.011501 | | |
|  | Lympho | | 4.666556 | | 1.572736 | | | 2.967158 | | | 0.003006 | | |
|  | CD4/CD8 | | -0.46062 | | 0.25391 | | | -1.81411 | | | 0.069662 | | |
|  | MLR | | 0.778003 | | 0.325363 | | | 2.391187 | | | 0.016794 | | |
|  | CD8T:Mono | | 0.308014 | | 0.108437 | | | 2.840478 | | | 0.004505 | | |
|  | Bcell:CD4/CD8 | | 0.417632 | | 0.195185 | | | 2.13968 | | | 0.032381 | | |
| The model based on data in the 5^th^ year | | | | | | | | | | | | |  |
|  | (Intercept) | | -1.60052 | | 0.110303 | | | -14.5102 | | | 1.04E-47 | | |
|  | mDI | | 0.343308 | | 0.114884 | | | 2.988298 | | | **0.002805** | | |
|  | CD8T | | -0.06192 | | 0.12104 | | | -0.51155 | | | 0.608963 | | |
|  | Mono | | 0.65959 | | 0.380669 | | | 1.732713 | | | 0.083147 | | |
|  | Gran | | 3.918209 | | 1.289601 | | | 3.038311 | | | 0.002379 | | |
|  | Lympho | | 4.689834 | | 1.354131 | | | 3.463353 | | | 0.000533 | | |
|  | MLR | | 0.760769 | | 0.302013 | | | 2.518997 | | | 0.011769 | | |
|  | CD8T:Mono | | 0.306931 | | 0.098065 | | | 3.129863 | | | 0.001749 | | |
| The model based on data in the 6^th^ year | | | | | | | | | | | | |  |
|  | (Intercept) | | -1.4524 | | 0.111412 | | | -13.0363 | | | 7.61E-39 | | |
|  | mDI | | 0.523538 | | 0.123888 | | | 4.225891 | | | **2.38E-05** | | |
|  | CD8T | | -0.04178 | | 0.17157 | | | -0.24349 | | | 0.807624 | | |
|  | Mono | | 0.736459 | | 0.368972 | | | 1.995978 | | | 0.045936 | | |
|  | Gran | | 3.833902 | | 1.236935 | | | 3.099518 | | | 0.001938 | | |
|  | Lympho | | 4.427297 | | 1.293407 | | | 3.422973 | | | 0.000619 | | |
|  | CD4/CD8 | | -0.19655 | | 0.226514 | | | -0.86771 | | | 0.385552 | | |
|  | MLR | | 0.553154 | | 0.313035 | | | 1.767067 | | | 0.077217 | | |
|  | mDI:CD4/CD8 | | 0.367785 | | 0.188792 | | | 1.948102 | | | **0.051403** | | |
|  | CD8T:Mono | | 0.301957 | | 0.102647 | | | 2.941713 | | | 0.003264 | | |
| The model based on data in the 7^th^ year | | | | | | | | | | | | |  |
|  | (Intercept) | | -1.30813 | | 0.105964 | | | -12.3451 | | | 5.18E-35 | | |
|  | mDI | | 0.530419 | | 0.119305 | | | 4.445913 | | | **8.75E-06** | | |
|  | CD8T | | -0.10439 | | 0.166051 | | | -0.62864 | | | 0.529585 | | |
|  | Mono | | 0.9188 | | 0.317848 | | | 2.89069 | | | 0.003844 | | |
|  | Gran | | 3.356148 | | 1.172151 | | | 2.863238 | | | 0.004193 | | |
|  | Lympho | | 3.651856 | | 1.198766 | | | 3.046345 | | | 0.002316 | | |
|  | CD4/CD8 | | -0.27161 | | 0.223801 | | | -1.21364 | | | 0.224883 | | |
|  | mDI:CD4/CD8 | | 0.358337 | | 0.182417 | | | 1.964387 | | | **0.049485** | | |
|  | CD8T:Mono | | 0.233457 | | 0.095996 | | | 2.431956 | | | 0.015018 | | |
| The model based on data in the 8^th^ year | | | | | | | | | | | | |  |
|  | (Intercept) | | -1.13898 | | 0.096615 | | | -11.7888 | | | 4.46E-32 | | |
|  | mDI | | 0.473307 | | 0.106696 | | | 4.436012 | | | **9.16E-06** | | |
|  | CD8T | | 0.103286 | | 0.108641 | | | 0.950712 | | | 0.34175 | | |
|  | Mono | | 1.067654 | | 0.30832 | | | 3.462811 | | | 0.000535 | | |
|  | Gran | | 4.167178 | | 1.139802 | | | 3.656054 | | | 0.000256 | | |
|  | Lympho | | 4.312902 | | 1.171738 | | | 3.680774 | | | 0.000233 | | |
|  | CD8T:Mono | | 0.20631 | | 0.089744 | | | 2.298875 | | | 0.021512 | | |
| The model based on data in the 9^th^ year | | | | | | | | | | | | |  |
|  | (Intercept) | | -1.08334 | | 0.099944 | | | -10.8394 | | | 2.24E-27 | | |
|  | mDI | | 0.554958 | | 0.116824 | | | 4.750373 | | | **2.03E-06** | | |
|  | Mono | | 0.966118 | | 0.301499 | | | 3.20438 | | | 0.001354 | | |
|  | Gran | | 3.591748 | | 1.114735 | | | 3.222064 | | | 0.001273 | | |
|  | Lympho | | 3.768816 | | 1.137573 | | | 3.313032 | | | 0.000923 | | |
|  | CD4/CD8 | | -0.29524 | | 0.153131 | | | -1.92804 | | | 0.05385 | | |
|  | mDI:CD4/CD8 | | 0.483007 | | 0.180957 | | | 2.669184 | | | **0.007604** | | |
| The model based on data in the 10^th^ year | | | | | | | | | | | | |  |
|  | (Intercept) | | -1.02927 | | 0.105029 | | | -9.79982 | | | 1.13E-22 | | |
|  | mDI | | 0.549409 | | 0.119434 | | | 4.600096 | | | **4.22E-06** | | |
|  | Mono | | 1.095008 | | 0.301694 | | | 3.62953 | | | 0.000284 | | |
|  | Gran | | 4.02405 | | 1.11088 | | | 3.622398 | | | 0.000292 | | |
|  | Lympho | | 4.142071 | | 1.165355 | | | 3.554344 | | | 0.000379 | | |
|  | CD4/CD8 | | -0.42396 | | 0.174677 | | | -2.42711 | | | 0.01522 | | |
|  | NLR | | -0.17027 | | 0.323303 | | | -0.52666 | | | 0.598432 | | |
|  | mDI:CD4/CD8 | | 0.565295 | | 0.195668 | | | 2.88905 | | | **0.003864** | | |
|  | CD4/CD8:NLR | | -0.32856 | | 0.196995 | | | -1.66787 | | | 0.095341 | | |
| The model based on data in the 11^th^ year | | | | | | | | | | | | |  |
|  | (Intercept) | | -0.92974 | | 0.104603 | | | -8.88825 | | | 6.21E-19 | | |
|  | mDI | | 0.603748 | | 0.119972 | | | 5.032423 | | | **4.84E-07** | | |
|  | Mono | | 1.1239 | | 0.2997 | | | 3.750082 | | | 0.000177 | | |
|  | Gran | | 4.403038 | | 1.106558 | | | 3.979038 | | | 6.92E-05 | | |
|  | Lympho | | 4.500122 | | 1.16021 | | | 3.878713 | | | 0.000105 | | |
|  | CD4/CD8 | | -0.47818 | | 0.176673 | | | -2.70656 | | | 0.006798 | | |
|  | NLR | | -0.2074 | | 0.32271 | | | -0.64268 | | | 0.52043 | | |
|  | mDI:CD4/CD8 | | 0.55789 | | 0.197614 | | | 2.823132 | | | **0.004756** | | |
|  | CD4/CD8:NLR | | -0.3372 | | 0.201383 | | | -1.67443 | | | 0.094047 | | |
| The model based on data in the 12^th^ year | | | | | | | | | | | | |  |
|  | (Intercept) | | -0.5521 | | 0.092588 | | | -5.96301 | | | 2.48E-09 | | |
|  | mDI | | 0.441697 | | 0.09795 | | | 4.509428 | | | **6.5E-06** | | |
|  | CD8T | | 0.432954 | | 0.105737 | | | 4.094646 | | | 4.23E-05 | | |
|  | Gran | | 2.75877 | | 0.717465 | | | 3.845163 | | | 0.00012 | | |
|  | Lympho | | 3.255476 | | 0.879484 | | | 3.701573 | | | 0.000214 | | |
|  | MLR | | 1.114228 | | 0.300115 | | | 3.712672 | | | 0.000205 | | |
|  | mDI:MLR | | -0.20047 | | 0.100264 | | | -1.99943 | | | **0.045562** | | |
|  | CD8T:MLR | | 0.444239 | | 0.113553 | | | 3.912171 | | | 9.15E-05 | | |

**Table S4.** Parameters for the regression model for breast cancer at the 11^th^ year after adding age. * denotes p<0.05, ** denotes p<0.005, *** denotes p<0.001.

|  | Estimate | Std. Error | t value | Pr(>\|t\|) |  |
| --- | --- | --- | --- | --- | --- |
| (Intercept) | -4.31465 | 2.627496 | -1.64211 | 0.101050902 |  |
| mDI | -0.56993 | 0.168762 | -3.3771 | 0.000776311 | *** |
| Mono | 9.328818 | 2.703332 | 3.450859 | 0.000595044 | *** |
| Gran | 4.893259 | 2.543199 | 1.924057 | 0.054784469 |  |
| Lympho | 7.179176 | 2.507712 | 2.86284 | 0.004334071 | ** |
| CD4CD8 | -0.42897 | 0.201168 | -2.13242 | 0.033347106 | * |
| NLR | 0.170357 | 0.078605 | 2.167262 | 0.030577861 | * |
| age | -0.00132 | 0.002537 | -0.51908 | 0.603879481 |  |
| mDI:CD4CD8 | 0.143908 | 0.061451 | 2.341827 | 0.019491073 | * |
| CD4CD8: NLR | -0.00406 | 0.011312 | -0.35863 | 0.719985643 |  |

**Table S5.** The difference of explained variance of the regression model for breast cancer at the 11^th^ year after removing mDI. The full model included all variables from step-wise regression. The reduced model excluded mDI and mDI related interactions from the full model. AIC: Akaike Information Criterion, BIC: Bayesian Information Criterion.

|  | AIC | BIC | Bayes.factor | P | R square |
| --- | --- | --- | --- | --- | --- |
| Full model | 833.875 | 878.783 | 0.421 | 0.004 | 0.084 |
| Reduced model | 841.125 | 877.051 | 2.377 |  | 0.068 |

Figure S1. Tissue specificity of the 426 genes for mDI using gene2func (53 specific tissues) of GTEx v8. Significantly enriched DEG sets (P_Bonferroni_<0.05) are highlighted in red.


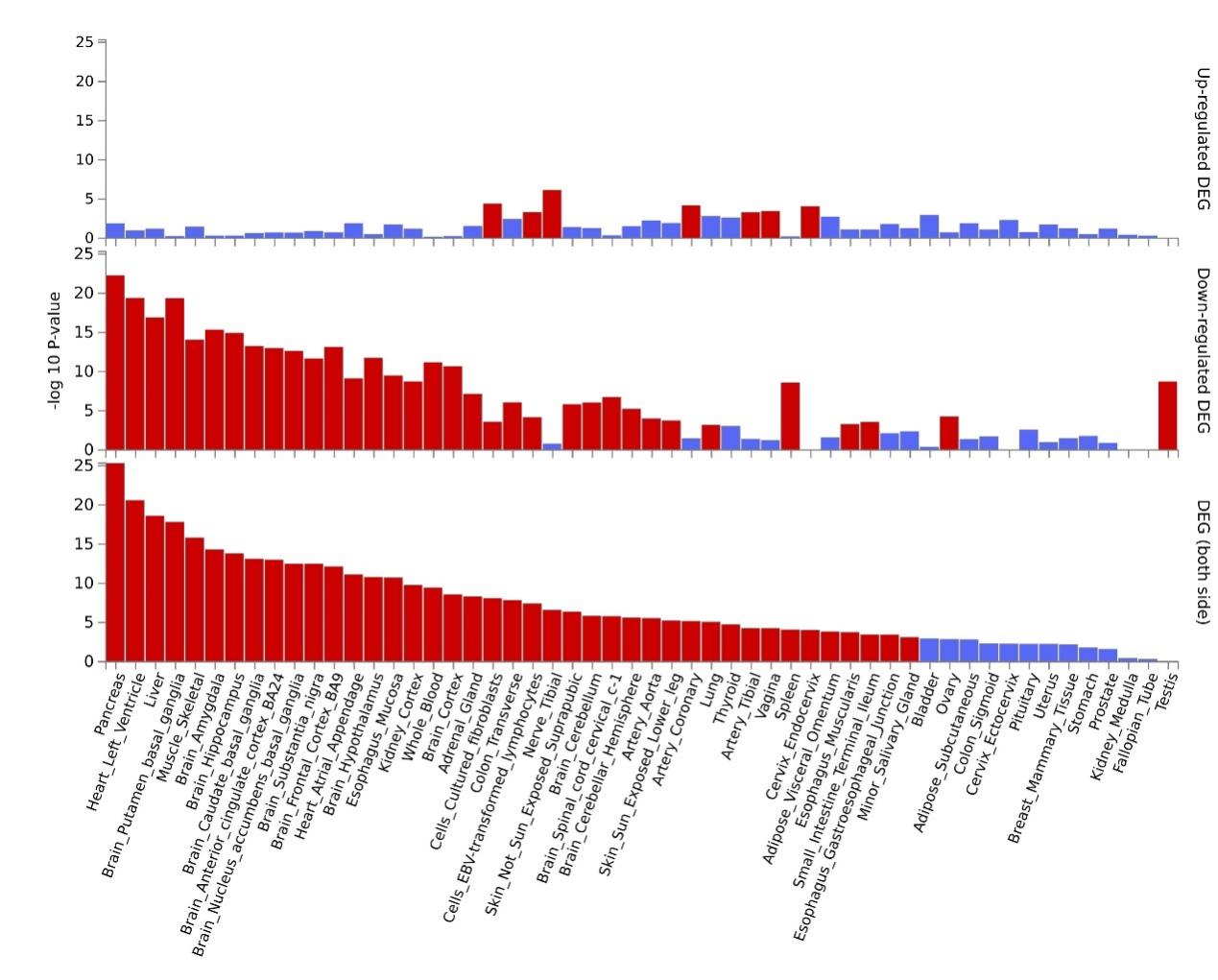

Supplement: Supplementary file 1 [file Table_1.DOCX]
